# Embedded Ileal Fish Bone Removed via Deep Enteroscopy in a Patient with Abdominal Pain and Hematochezia: A Case Report

**DOI:** 10.3390/medicina61010030

**Published:** 2024-12-28

**Authors:** Hsin-Yang Chen, Chao-Feng Chang, Tien-Yu Huang, I-Hsuan Huang

**Affiliations:** Division of Gastroenterology, Department of Medicine, Tri-Service General Hospital, National Defense Medical Center, No. 325, Section 2, Chenggong Road, Neihu District, Taipei City 114202, Taiwan; asadagoat@gmail.com (H.-Y.C.); taiwanvincent777@gmail.com (C.-F.C.); tienyu27@gmail.com (T.-Y.H.)

**Keywords:** fish bone, foreign body, abdominal pain, gastrointestinal tract bleeding, hematochezia, balloon enteroscopy, deep enteroscopy

## Abstract

Ingestion of foreign bodies is a prevalent issue in clinical practice, with fish bones being the predominant cause. While the upper gastrointestinal tract is commonly affected, small intestine impactions pose significant diagnostic challenges due to nonspecific symptoms and lack of awareness of foreign body ingestion. Herein, we describe a case presenting with recurrent, unexplained abdominal pain and hematochezia. Multiple diagnostic investigations, including esophagogastroduodenoscopy and colonoscopy, conducted over several months failed to identify the underlying cause until a retrograde single-balloon enteroscopy for obscure gastrointestinal bleeding revealed a 2.3 cm fish bone embedded in the distal ileum. The successful removal of the fish bone led to the resolution of the patient’s symptoms. This case highlights that foreign bodies in the small intestine can be a cause of hematochezia and emphasizes the growing importance of deep enteroscopy techniques in detecting and retrieving these foreign objects, thereby reducing the need for surgery.

## 1. Introduction

Ingestion of foreign bodies is a frequently encountered clinical problem [1]. Fish bones are reported to constitute the majority of foreign bodies in the upper digestive tract, especially in Asian countries, representing approximately 50% to 90% of all cases [2]. In comparison to oropharyngeal fish bones, which are more easily detected and removed by endoscopy, fish bones located in the small intestine pose significant diagnostic challenges. These challenges arise due to the nonspecific symptoms and the difficulty in obtaining an accurate history after a period of time following accidental ingestion of foreign bodies [3].

In this report, we describe a patient who presented with recurrent abdominal cramps and bloody stools. She had several hospital visits before the cause, a fish bone embedded in the distal ileum, was identified. The patient underwent retrograde single-balloon enteroscopy, during which the foreign body was identified and successfully removed. To our knowledge, there are few reports in the literature describing fish bone removal via deep enteroscopy. Our case underscores the effectiveness of balloon-assisted enteroscopy in reducing the need for further invasive surgical interventions, particularly in uncomplicated cases.

## 2. Case Presentation

**Figure 1:**
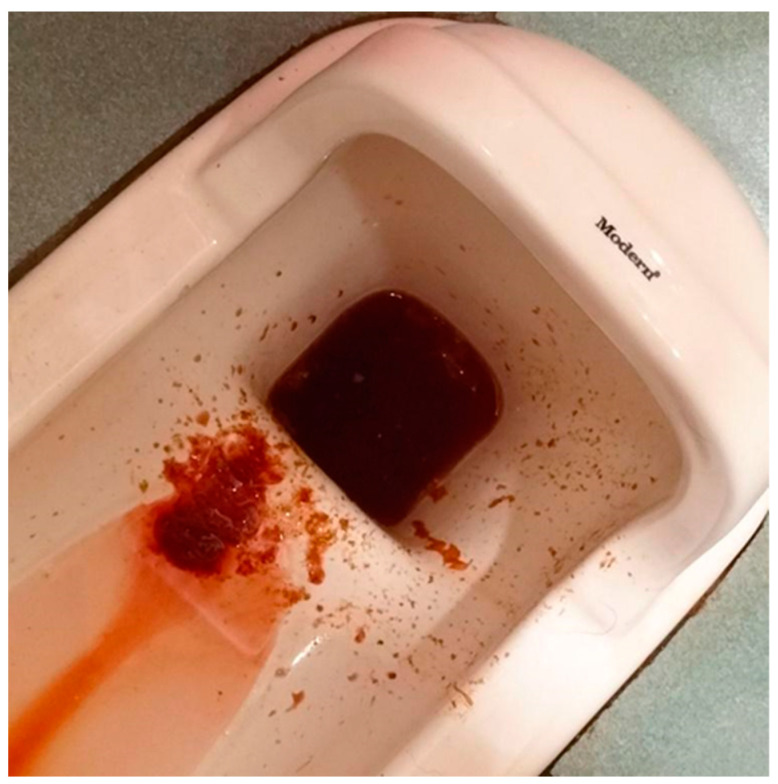
This is a photo of hematochezia before admission.

A 51-year-old woman presented with intermittent lower abdominal cramping and hematochezia over the past several months. She had a medical history of thalassemia, and she had surgical history of cholecystectomy. She reported no use of NSAIDs or antiplatelet or anticoagulant agents. The patient had consulted multiple hospitals over the course of months and underwent multiple diagnostic investigations, including abdominal computed tomography scans, esophagogastroduodenoscopy, and colonoscopy. These investigations failed to identify any significant mucosal lesion or source of bleeding. The stool pathogen survey yielded no evidence of infection. After conservative treatment, the patient experienced temporary symptom relief. However, another episode of intermittent lower abdominal cramping with bloody diarrhea occurred (Figure 1).

She then presented to our hospital for further evaluation. Her vital signs on admission included a blood pressure of 125/81 mmHg, body temperature of 36.8 °C, respiratory rate of 16 breaths per minute, and an oxygen saturation (SpO2) of 98% on room air. Physical examination revealed a soft and non-distended abdomen, increased bowel sounds, and lower abdominal tenderness without rebound pain or muscular guarding. Laboratory findings were predominantly normal, except for microcytic anemia (hemoglobin at 11.1 g/dL, mean corpuscular volume [MCV] at 58.8 fL), mildly elevated C-reactive protein levels (1.23 mg/dL), and a positive result on the stool occult blood test (4+).

**Figure 2:**
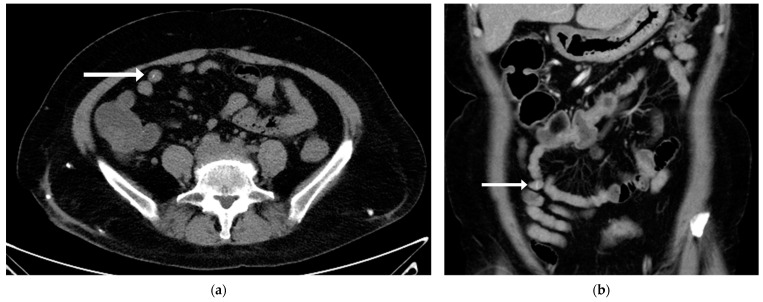
The abdominal computed tomography (CT) revealed a linear hyperdense content (fish bone) within the terminal ileum. (**a**) The axial view of the non-contrast abdominal CT showed a continuous hyperdense spot within the ileum, indicated by the arrow. (**b**) The coronal view of the contrast-enhanced abdominal CT demonstrated a linear hyperdense object consistent with the axial view, indicated by the arrow.

Following admission, the patient experienced frequent episodes of lower abdominal cramping pain accompanied by bloody diarrhea. Her hemoglobin decreased from 11.1 g/dL to 8.8 g/dL, prompting repeat esophagogastroduodenoscopy and colonoscopy. No significant sources of bleeding or lesions were detected in the gastric, duodenal, or colonic mucosa. Suspecting obscure gastrointestinal bleeding, we conducted a series of examinations, including repeated abdominal computed tomography with angiography and nuclear medicine gastrointestinal bleeding scan. Despite these examinations, no contrast extravasation and no significant bleeding source were identified. To further investigate the obscure gastrointestinal bleeding and to evaluate the abdominal pain of unclear etiology, capsule endoscopy was performed. However, no mucosal abnormality or apparent lesion was detected. The linear hyperdense lesion on the abdominal CT was identified only after a detailed reassessment of the images, following the failure of multiple diagnostic attempts (Figure 2).

**Figure 3:**
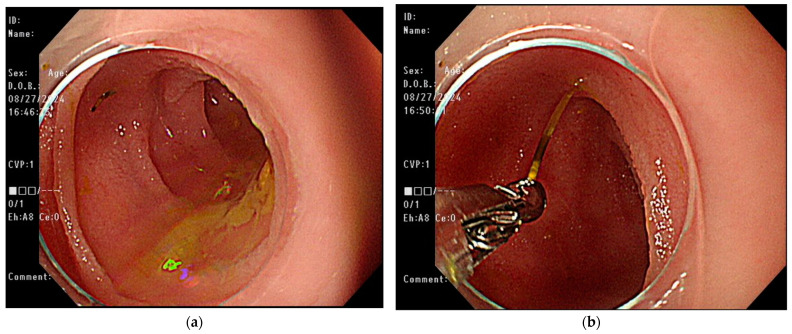
Endoscopic finding of retrograde single-balloon enteroscopy. (**a**) A sharp foreign body was embedded in the distal ileum. (**b**) The foreign body was successfully removed with forceps.

**Figure 4:**
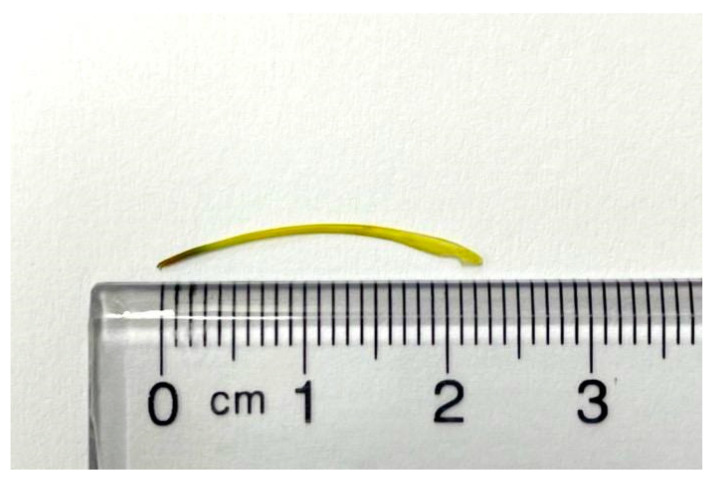
Photograph of the yellow, sharp foreign body, approximately 2.3 cm in length. The foreign body was confirmed to be a fish bone upon pathological examination.

The patient continued to experience abdominal cramping and intermittent episodes of bloody diarrhea despite receiving conservative treatment. The persistent symptoms necessitated the decision to pursue more invasive diagnostic procedures. Given the presence of obscure intestinal bleeding and a suspected linear lesion in the small intestine, retrograde single-balloon enteroscopy was performed. During the procedure, a sharp foreign body embedded in the distal ileum was identified, and it was successfully removed by endoscopic forceps (Figure 3). After the procedure, the patient’s unexplained abdominal pain with intermittent bloody diarrhea resolved at subsequent clinic follow-ups. The yellow, sharp foreign body was about 2.3 cm in length and was confirmed to be a fish bone upon pathological examination (Figure 4).

## 3. Discussion

Foreign body ingestion and food bolus impaction are frequently encountered in clinical practice worldwide [1]. The primary causes of foreign body ingestion are influenced by age, underlying conditions, and cultural or geographic factors. Adults with psychiatric illnesses or impaired mental status (such as developmental disabilities or substance intoxication), and certain populations like prisoners or drug traffickers have an increased risk for true foreign body ingestion [4,5]. In cases of foodborne foreign body disease, esophageal bolus impaction is the most prevalent type, with an estimated incidence of 13–16 per 100,000 persons per year [4,5]. In contrast, the prevalence rate of foreign bodies in the intestine remains unclear.

Fish bone impaction remains the most common type of foreign body ingestion in Asian countries, especially in coastal areas [2,5,6]. Most ingested fish bones are passed uneventfully, while the minority require endoscopic removal [3,6,7]. Only 1% perforate or penetrate the gastrointestinal tract, potentially migrating to organs such as the liver and pancreas, and thus requiring surgical intervention [8,9]. Entrapment in the upper aerodigestive tract is the most frequent complication following the ingestion of fish bones. Common lodging sites in the oropharynx include the tonsils, base of the tongue, vallecula, pyriform sinus, and posterior wall of the hypopharynx [2,7]. Major complications, such as perforation and bleeding, typically occur in areas of the gastrointestinal tract that are angulated or narrow [3]. In the esophagus, three areas of physiological narrowing—the upper esophageal sphincter, prominence of the aortic arch or the left main bronchus, and the lower esophageal sphincter—remain the most frequent sites of impaction [6]. Perforation of the esophagus by fish bones occurs in 1–4% of cases but can cause mediastinal abscess, surrounding vascular injuries, and fatal aortoesophageal fistula [3,10,11,12]. The ileum, ileocecal junction, and rectosigmoid colon are the most common sites of perforation within the bowel, which may require further surgical intervention [3,13,14,15,16,17].

The management of foreign bodies varies based on the type of object, but sharp objects should be promptly removed from the gastrointestinal tract. The European Society of Gastrointestinal Endoscopy (ESGE) advises that therapeutic endoscopy be performed urgently (within 24 h) for foreign bodies in the stomach or small intestine, including sharp-pointed objects, magnets, batteries, and large or long objects greater than 5–6 cm in length [1]. Fish bones lodged in the upper aerodigestive tract can be readily identified because they usually present as an acute event with a short history [3]. Patients are generally able to identify the correct location of the fish bone if it is lodged in the upper esophagus or higher [3]. However, diagnosing fish bone impaction below the esophagus is challenging due to the vague symptoms that complicate precise localization [6]. The symptoms of fish bone impaction below the esophagus, including abdominal pain, peritoneal irritation, bowel obstruction, and hemorrhage, often mimic other conditions, such as appendicitis, diverticulitis, colitis, peptic ulcer disease, or gastrointestinal bleeding upon initial clinical assessment [3,15,18,19]. Additionally, the onset of symptoms may be delayed, and patients may not recall having accidentally ingested foreign bodies [3]. The nonspecific clinical manifestations and lack of awareness of fish bone ingestion during history-taking complicate the immediate diagnosis of intestinal fish bone impaction, which may delay urgent endoscopic removal.

In some cases, fish bones become deeply embedded in the gastrointestinal tract, making them invisible and undetected during endoscopy. In such instances, the fish bone may be removed through endoscopic mucosal incision, guided by CT imaging and endoscopic findings [20]. In other cases, the fish bone migrates out of the aerodigestive tract or affects nearby organs, such as the liver, pancreas, or aorta. These cases are often managed through surgical intervention, such as laparoscopic surgery or exploratory laparotomy [8,9,13,14,16,17]. Laparoscopic surgery offers several advantages over laparotomy, including relatively minor trauma, less pain, quicker recovery, and a shorter duration of hospitalization. However, if there is significant abdominal inflammatory adhesion or if laparoscopic surgery proves unsuccessful, laparotomy should be considered as an alternative [21].

A few reports in the literature have described fish bone removal via deep enteroscopy (Table 1). In line with our case, Shibuya et al. reported a 33-year-old man experiencing persistent postprandial fullness [22]. He was previously diagnosed with functional dyspepsia, but did not respond to 8 months of medication treatment. He underwent an antegrade double-balloon enteroscopy, during which an 11 mm fish bone lodged in the jejunum was discovered. Removal of the fish bone by forceps led to a dramatic improvement in his abdominal symptoms. Alkhatib et al. described a 67-year-old male presenting with six days of dull abdominal pain localized to the left lower quadrant [23]. The abdominal CT scan showed mesenteric stranding over a loop of jejunum with a small amount of adjacent free air. Due to a suspected sealed perforation, the patient underwent double-balloon-assisted enteroscopy, which revealed a small superficial ulcer in the mid-jejunum with a fish bone protruding from the center of the ulcer. Following the successful removal of the foreign body using a snare, the patient recovered and was able to resume his regular diet the next day without the need for further surgical intervention. Shimozaki et al. presented a 49-year-old woman with upper abdominal pain [24]. The abdominal CT image revealed a linear, high-density structure in the proximal jejunum, which was identified as a fish bone. It was successfully removed using single-balloon endoscopy.

In our case, a sharp fish bone measuring approximately 2.3 cm in length was embedded in the distal ileum, nearly causing a perforation. The clinical presentation was nonspecific, characterized by intermittent lower abdominal pain with hematochezia. During the initial evaluation, neither the chief complaint nor the medical history indicated prior fish bone ingestion. Even after the diagnosis, the patient was unable to recall the timing of the ingestion. On subsequent abdominal CT, the linear hyperdense lesion was initially overlooked due to a lack of observer awareness. Additionally, in patients administered intravenous contrast, the linear bony lesions can resemble small blood vessels, further complicating timely diagnosis. The foreign body was only identified after a meticulous review of the abdominal CT images, following the failure of multiple diagnostic procedures. Among these procedures, the capsule enteroscopy failed to detect the fishbone embedded in the distal ileum. According to the literature, the overall detection rate of capsule enteroscopy is approximately 59%, with a detection rate of 55% for obscure gastrointestinal bleeding [25]. Despite its ability to provide a comprehensive examination of the majority of the small bowel mucosa, capsule endoscopy may fail to identify certain lesions, such as neoplasms [26]. The deep embedding of the fish bone, with minimal protrusion into the intestinal lumen, may obscure its detection during capsule endoscopy.

With advances in deep enteroscopy, the detection and removal of small intestinal foreign bodies have become more readily available. Reviews have documented successful retrieval of various objects through the use of balloon enteroscopy, including retained capsule endoscopies, dentures, medical tubes, metal stents, toothpicks, and fish bones [27,28]. In one review, medical devices (29/34 cases [85.3%]) constituted the majority of foreign bodies entrapped within the small intestine, and seventeen cases (50%) were successfully removed through balloon enteroscopy [27]. Only two patients (5.9%) developed procedure-related complications: one perforation during balloon dilation and one case of acute pancreatitis following the procedure [27]. Balloon enteroscopy serves as a safe and effective first-line approach for the diagnostic and therapeutic management of foreign bodies in the small intestine, particularly in uncomplicated situations.

## 4. Conclusions

Fish bones constitute a significant proportion of ingested foreign bodies, particularly in Asian countries. The most common impaction sites are in the upper aerodigestive tract, where they can be easily detected and removed endoscopically. The fish bone distal to the esophagus poses a diagnostic and therapeutic challenge due to nonspecific symptoms and unawareness of fish bone ingestion. Our case highlights the importance of considering gastrointestinal foreign bodies in patients presenting with persistent unexplained abdominal symptoms. Advances in deep enteroscopy have improved the detection and management of small intestinal foreign bodies, diminishing the reliance on surgical intervention and establishing balloon enteroscopy as a safe and effective treatment option.

## Figures and Tables

**Table 1:** Summary of cases involving fish bone impaction in the small intestine successfully treated with balloon enteroscopy.

Author	Patient	Symptoms	Impaction Site	Management	Fish Bone Length	Outcome
Shibuya et al. [22]	33/M	Postprandial discomfort in the upper abdomen	Jejunum	Antegrade DBE with forceps	1.1 cm	Resolution of symptoms
Alkhatib et al. [23]	67/M	LLQ dull abdominal pain, nausea, chills	Mid-jejunum	Antegrade DBE with a snare	2.2 cm	Resolution of symptoms
Shimozaki et al. [24]	49/F	Upper abdominal pain	Proximal jejunum	Antegrade SBE with forceps	N/A	Resolution of symptoms
Our case	51/F	Lower abdominal cramping pain, hematochezia	Distal ileum	Retrograde SBE with forceps	2.3 cm	Resolution of symptoms

DBE: double-balloon endoscopy, SBE: single-balloon endoscopy.

## Data Availability

The data presented in this study are available from the corresponding author upon reasonable request.
